# Barriers to COVID-19 vaccination among older adults in Mexico City

**DOI:** 10.1186/s12939-022-01685-6

**Published:** 2022-06-19

**Authors:** Pablo Gaitán-Rossi, Miranda Mendez-Rosenzweig, Erika García-Alberto, Mireya Vilar-Compte

**Affiliations:** 1 Research Institute for Equitable Development, EQUIDE, Universidad Iberoamericana, Prolongación Paseo de la Reforma 880, Lomas de Santa Fe, 01219 Mexico City, Mexico; 2 Department of Public Health, Montclair State University, University Hall 4157, 1 Normal Ave, Montclair, NJ 07043 USA

**Keywords:** COVID-19, Vaccine hesitancy, Older adults, Food insecurity, Health inequities

## Abstract

Vaccines are effective to reduce COVID-19 related outcomes, but universal vaccination campaigns can reveal within-country access inequities. Mexico City has had high rates of COVID-19 related morbidity and mortality and a population survey warned that vaccine acceptance was lowest in older adults. Since February 2021, Mexico started a universal and free vaccination campaign prioritizing older adults. By April 17, every older adult in Mexico City had been eligible to receive the first dose. A week later, we conducted a telephone survey representative of older adults residing in Mexico City (*n* = 503). We asked if they received their first dose and, if they haven’t, we followed-up with an open question to register their reasons. In addition to sociodemographic characteristics and food insecurity, we also inquired about vaccine hesitancy, health concerns related to COVID-19, self-rated health, comorbidities, frailty, and depression. The objective of the study was to identify the main barriers to receive the first dose of the vaccine. We estimated descriptive statistics and logistic regression models. Results show that 7.6% of older adults in Mexico City did not receive their first dose. Barriers for not receiving it were vaccine hesitancy (60.4%), not having COVID-19 health concerns (46.4%), poor self-rated health (46.7%), a previous diagnosis of depression (35.7%), low socioeconomic status (65.4%), and household food insecurity (59.8%). Responses to the open question clustered in four themes: misinformation about the process (30%), distrust of the vaccine (24%), personal health problems (24%), and difficulties to get an appointment (22%). Logistic regression models adjusted for vaccine hesitancy and revealed two distinct reasons for not having their first dose: 1) vaccine hesitancy and misinformation on COVID-19, and 2) household food insecurity. Reaching these two groups requires active and differentiated public-health measures; the first with additional information from trusted sources, and the second by facilitating vaccination in neighborhoods with high levels of food insecurity and informal labor, where missing a day’s work is a strong disincentive. Vaccination campaigns need an equity lens to reach universal coverage; ensuring full access demands thorough and carefully tailored new interventions.

**Supplementary Information:**

The online version contains supplementary material available at 10.1186/s12939-022-01685-6.

## Background

Vaccines are a powerful tool to abate COVID-19 related outcomes [1]. However, vaccination campaigns have revealed important health inequities between and within countries [2]. Vaccination data from middle-and low-income countries has been scarce, and it is important to identify groups without sufficient access to vaccines to design supplementary strategies to reach them [3].

## Survey and results

Mexico has been one of the most affected countries in the world by the pandemic and Mexico City had the highest rates of morbidity and mortality in the country [4]. After vaccinating COVID-19 first-responders in December 2020, Mexico started a universal and free vaccination campaign in February 2021 [5]. One of the main criterions to prioritize access was by age: adults 60 years or older were the first to receive the shots. Older adults in Mexico City registered on a webpage, received an SMS message with their appointment, and then went to vaccination centers located in their municipality [5]. Persons unable to register online received assistance at the vaccination center. The only mandatory documents were an official identification to ensure age-group and evidence of place of residence to validate municipality. Those unable to attend the centers could opt for in-house vaccination. There were no reports of vaccine scarcity, and, at the time, vaccines were available for this age-group. Nonetheless, the federal government allotted four different brands of vaccine –Sinovac, Pfizer, AstraZeneca, or Sputnik– by municipality (i.e., within municipality, everyone received the same brand). Mexico City’s government announced on April 17 that 83% of the older adults already had at least the first dose [6]. By June 22, 92% of older adults in Mexico City had at least one dose, while 88% were fully vaccinated [7].

Before the pandemic, a meta-analysis on vaccine uptake found older adults were more likely to accept one, while education and income had mixed results when vaccines were free [8]. A study in the United Kingdom about the COVID-19 vaccine found uptake was generally high and hesitancy concentrated on females and subgroups characterized by lower education and some ethnic minorities [9]; a relatively low proportion of older adults (4.1–14.3%) expressed it was unlikely they will receive the shot. In Mexico, even though older adults have a higher risk of COVID-19 mortality, a population study conducted between September and November 2020 found that older adults –especially female– had the lowest vaccine acceptance between age-groups (54.0%); acceptance increased with education and having formal employment, and Mexico City was the state with the highest percentage of vaccine acceptance (69.3%), still low in comparison with other countries [10].

Our study explores associations between vaccine hesitancy and other risk factors among the older adults in Mexico City to help explain who has not received the first dose of the COVID-19 vaccine. Between April 22 and May 20, 2021, we conducted a telephone survey representative of older adults residing in Mexico City. We used a probabilistic stratified single-stage sampling of landline and mobile telephone numbers; see additional details and the distribution of sample by municipality in Fig. 1 in the Supplementary material. Persons who reported problems with their memory were excluded. The analytic sample size was 503 eligible older adults; with respondents from each of the 16 boroughs in Mexico City, 57% female, 55% between 60 and 70 years old, and 50% middle-low socioeconomic status (SES).

We asked if they had received their first dose of the COVID-19 vaccine. A negative response prompted a follow-up question on the reasons why they did not get it. In addition, we asked about vaccine hesitancy (“How safe do you consider COVID-19 vaccines?”); COVID-19 health concerns (“How worried are you that your health might be affected by COVID-19”); comorbidities; self-reported health; frailty (FRAIL scale), depression (CESD-7), food insecurity (FIES) and sociodemographic characteristics. Lastly, we paired municipality of residence with the brand of vaccine assigned to each municipality.

We conducted bivariate descriptive statistics to identify the salient characteristics of those without a vaccine and estimated logistic regressions to adjust associations. All analyses used sampling weights. Tables with results and measurement details are in the supplementary material. The questionnaire, a methodological note, and dataset are publicly available. The key limitation of the study is having a small sample size to identify risk factors for a rare phenomenon in the target population.

Our findings approximate official estimates: despite being eligible, 7.6% of the older adults in Mexico City reported not having their first dose of the vaccine. The majority considered the vaccines were safe (72.9%). Importantly, one-fourth of the vaccinated overcame their hesitancy to receive the shot. The variables associated with not-having received their first dose were vaccine hesitancy (60.4%), not having COVID-19 health concerns (46.4%), poor self-rated health (46.7%), a previous diagnosis of depression (35.7%), low socioeconomic status (65.4%), and household food insecurity (59.8%). The analysis of the open question on the reasons why they don’t have the vaccines yielded four main themes: misinformation about the vaccination process (30%), distrust of the vaccine (24%), personal health problems (24%), and lack of geographical and/or temporal access at the time of their appointment (22%).

Logistic models continued to explore the associations between vaccine hesitancy and other relevant variables. Vaccine hesitancy remained statistically significant when tested against each covariate –including its interactions (not shown). Not having COVID-19 concerns was significant even when considering vaccine hesitancy (OR = 2.67), indicating that misinformation about the diseases is in itself a driver that prevents vaccination. Vaccine brand was not associated with uptake. The health variables –self-reported health, comorbidities, and a diagnosis of depression–ceased to be associated with vaccination once vaccine hesitancy was accounted for. Therefore, health problems are probably a weak explanation for the refusal to get the vaccine amongst older adults in Mexico City. Even though SES was not statistically significant in the models, food insecurity (OR = 2.58) kept being associated with not having the first dose of the vaccine.

Our results exemplify that free and universal vaccination campaigns that require assistance to medical centers do not reach all groups. Even though every older adult in Mexico City has been eligible to receive the vaccine, since September 2021 the coverage stubbornly remains at 94% of fully vaccinated older adults in Mexico City [11]. Currently, there is no publicly available data on vaccination to explain who is that 6% and why they did not receive the vaccine. Our results suggest vaccine hesitancy and COVID-19 misinformation are key reasons why older adults in Mexico City might be refusing the vaccine. In addition, our study shows there might be a second group of unvaccinated older adults in Mexico City, namely, those with food insecurity in the household. A limitation of the study is that we did not explore if the second group is also associated with time availability and hours worked by day. Further studies deepening on socioeconomic status need to examine if, indeed, the association can be explained by the need of daily income for basic needs because it would indicate wider schedules to get the vaccine are warranted.

## Conclusions

Universal coverage in vaccination campaigns requires an equity lens. Active and differentiated public-health measures targeting hard-to-reach populations are needed because these two groups might not even request the vaccine [12]. The first group could be tackled with additional information from trusted sources. The second group might need to approximate vaccination centers to neighborhoods with high levels of food insecurity and informal labor, where missing a day’s work is a strong disincentive to get the shot. At the beginning of the campaign, high demand facilitates vaccination, however, ensuring full access needs thorough and carefully tailored new interventions.

## Supplementary Information

**Additional file 1.**

## Data Availability

The data is publicly available: Gaitán-Rossi, Pablo, Mendez-Rosenzweig, Miranda, García-Albertos, Erika, & Vilar-Compte, Mireya. (2022). Encuesta de Salud y Alimentación de Adultos Mayores de la Ciudad de México 2021 (ENSAAM) (One) [Data set]. Zenodo. 10.5281/zenodo.6522672.

## Abbreviations

SMS: (Short Message Service)
CESD-7: (Center for Epidemiologic Studies Depression Scale)
FIES: (Food Insecurity Experience Scale)
SES: (Socioeconomic Status)
OR: (Odds Ratio)
