# Supplementary material for: Barriers to COVID-19 vaccination among older adults in Mexico City
Source: Int J Equity Health. 2022 Jun 19;21:85. doi: 10.1186/s12939-022-01685-6 (PMC9206538; doi:10.1186/s12939-022-01685-6)
Supplement: Supplementary file 1 — Additional file 1. [file 12939_2022_1685_MOESM1_ESM.docx]

**SUPPLEMENTARY MATERIAL**

Barriers to COVID-19 vaccination among older adults in Mexico City

**Figure 1**. Comparison of the proportions between population size of older adults, weighted sample, and unweighted sample from the ENSAAM survey by Mexico City municipalities.


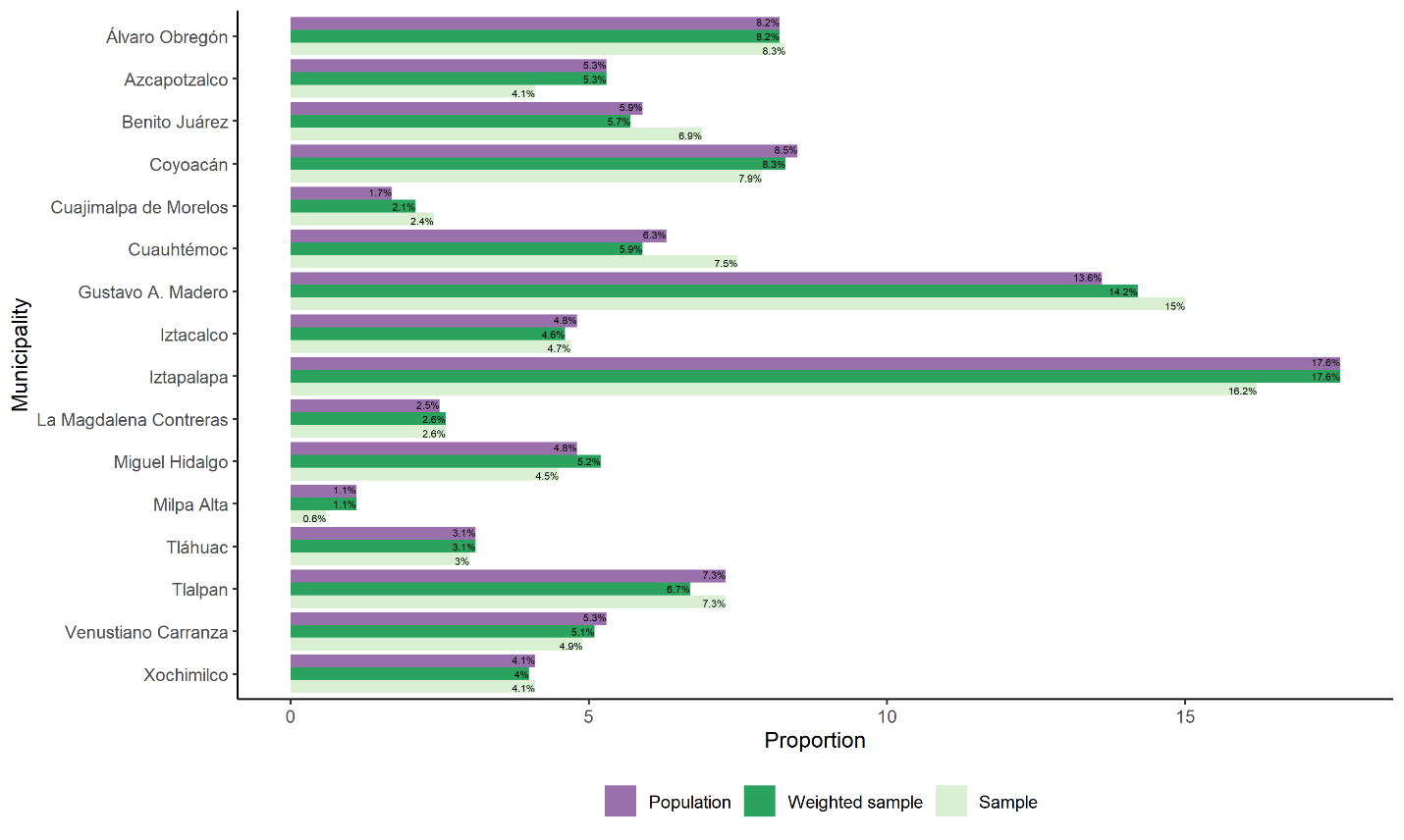


*Note*: The sampling design for the ENSAAM survey was a stratified one-stage probabilistic sample of fixed and mobile telephone numbers, implemented by 'Random Digit Dialing' (RDD). Stratification was done by municipality and proportions were obtained from the 2020 Census according to the distribution of people aged 60 and over in Mexico City. The sample frame was the National Dialing Plan from the Instituto Federal de Telecomunicaciones (Mexico’s Federal Institute of Telecommunications, IFT); updated as of February 11, 2021. Sampling weights were estimated based on municipality strata and sociodemographic domains using the distribution of the target population, also from the 2020 Census; domains considered were age, sex, education of head of household, and internet available in household.

**Table 1**. Descriptive statistics and bivariate associations between Covid-19 Vaccination and socioeconomic and health characteristics.

|  | **Total** | **% (n)** | **% (n)** | **P-value** |  |
| --- | --- | --- | --- | --- | --- |
|  |  |  |  |  |  |
| **COVID-19 Vaccination** |  | No | Yes |  |  |
|  | 100% (503) | 7.63% (38) | 92.38% (465) |  |  |
| **Gender** |  | No | Yes | 0.4502 |  |
| Females | 56.71% (278) | 62.59% (23) | 56.23% (255) |  |  |
| Males | 43.29% (225) | 37.41% (15) | 43.77% (210) |  |  |
| **Age** |  | No | Yes | 0.3418 |  |
| 60 to 69 | 55.14% (299) | 47.60% (20) | 55.76% (279) |  |  |
| 70 & more | 44.86% (204) | 52.40% (18) | 44.24% (186) |  |  |
| **Socioeconomic Status (SES)** |  | No | Yes | 0.0671 |  |
| Middle-low | 50.03% (226) | 65.43% (21) | 48.83% (205) |  |  |
| Middle-high | 49.97% (239) | 34.57% (12) | 51.17% (227) |  |  |
| **Vaccine Safety** |  | No | Yes | 0.0000 |  |
| Regular / Unsafe | 27.09% (129) | 60.37% (18) | 24.82% (111) |  |  |
| Safe | 72.91% (343) | 39.63% (12) | 75.18% (331) |  |  |
| **COVID-19 vaccine brand** |  | No | Yes | 0.4358 |  |
| CoronaVac-Sinovac | 11.74% (61) | 11.52% (5) | 11.76% (56) |  |  |
| Pfizer/BioNTech | 19.05% (84) | 8.91% (3) | 19.89% (81) |  |  |
| Gamaleya-Sputnik | 43.49% (216) | 48.58% (18) | 43.07% (198) |  |  |
| Oxford/AstraZeneca | 25.71% (142) | 31% (12) | 25.27% (130) |  |  |
| **COVID-19 health concerns** |  | No | Yes | 0.0204 |  |
| Not worried | 29.19% (142) | 46.35% (16) | 27.80% (126) |  |  |
| Worried | 70.81% (356) | 53.65% (21) | 72.20% (335) |  |  |
| **Comorbidities (ever diagnosed)** |  | No | Yes | 0.7142 |  |
| At least one | 75.95% (378) | 78.51% (30) | 75.74% (348) |  |  |
| None | 24.05% (123) | 21.49% (8) | 24.26% (115) |  |  |
| **Self-reported health** |  | No | Yes | 0.0045 |  |
| Average / Poor / Very bad | 26.56% (130) | 46.70% (17) | 24.92% (113) |  |  |
| Good / Very good | 73.44% (369) | 53.30% (20) | 75.08% (349) |  |  |
| **Frailty (FRAIL Scale)** |  | No | Yes | 0.6113 |  |
| Pre-frailty & frailty | 52.18% (259) | 48.12% (18) | 52.52% (241) |  |  |
| No frailty | 47.82% (244) | 51.88% (20) | 47.48% (224) |  |  |
| **Depression (ever diagnosed)** |  | No | Yes | 0.0094 |  |
| Yes | 19.02% (93) | 35.71% (13) | 17.72% (80) |  |  |
| No | 80.98% (401) | 64.29% (23) | 82.28% (378) |  |  |
| **Depression Scale (CESD-7)** |  | No | Yes | 0.8491 |  |
| Symptoms | 35.47% (171) | 33.97% (12) | 35.58% (159) |  |  |
| No symptoms | 64.53% (316) | 66.03% (23) | 64.42% (293) |  |  |
| **Food Insecurity Experience Scale (FIES)** |  | No | Yes | 0.0237 |  |
| Food Insecurity | 41.25% (189) | 59.83% (21) | 39.72% (168) |  |  |
| Food Security | 58.75% (280) | 40.17% (14) | 60.28% (266) |  |  |

*NOTE*: Percentages estimated with sampling weights. All statistical tests are chi-squared hypothesis tests with a confidence interval of 95%. *COVID-19 vaccination* is the Yes/No answer to the question: “Did you receive the COVID-19 vaccine?”. *Gender* is a dichotomous question Male/Female. *Age* in years, recoded into two groups: 60-69 years old and 70 or more (max value= 97). *SES*, Socioeconomic Status, measured with the validated 5-item assets-based AMAI index (Asociación Mexicana de Agencias de Mercado de México) and recoded from the original seven levels (A/B, C +, C, C-, D +, D or E) to two groups: Middle-high SES (C/C+/A/B) and Middle-low SES (E/D/D+/C). *Vaccine safety*, “How safe do you consider COVID-19 vaccines?”, was recoded from three response options to “Safe” and “Regular/Unsafe”. *COVID-19 health concerns*, “How worried are you that your health might be affected by COVID-19”, was recoded as “no-worried” or “worried” (little worried/ somewhat worried/ very worried). *Comorbidities*, ever diagnosed on: Diabetes, High Cholesterol, Hypertension, Heart condition, Cancer, Asthma or bronchitis, Depression, Arthritis, Hepatic condition or cirrhosis, Osteoporosis, Kidney conditions, Gastritis. *Self-reported health,* “How is your health status today?”, recoded from 5 response options to two: “Good / Very good” and “Average/Poor/ Very bad”. *Frailty*, assessed with the 5-item FRAIL scale: fatigue, resistance, ambulation, illnesses, and weight loss (the summative scores were re-coded O for “No Frailty” and 1 – 5 as “Pre-Frailty and Frailty”). *Depression* ever diagnosed depression from the list of comorbidities. *Depression Scale*, assessed with the 7-item CESD scale (Center for Epidemiologic Studies Depression) with a cutoff score of ≤4 points, re-coded as “Symptoms” and “No symptoms”. *Food Security*, assessed with the 8-item Food Insecurity Experience Scale (FIES) with a cutoff score of ≤1 points.

Table 2. Logistic regression Models adjusted by vaccine hesitancy (sample= 503).

|  | (1) | (2) | (3) | (4) | (5) | (6) | (7) | (8) |
| --- | --- | --- | --- | --- | --- | --- | --- | --- |
| VARIABLES | Vaccine hesitancy | Vaccine brand | Health concerns | Self-reported health | Comorbidities | Depression | SES | Food Insecurity |
|  |  |  |  |  |  |  |  |  |
| Vaccine hesitancy: Unsafe (Ref. group: Safe) | 4.613** | 4.748** | 5.355** | 4.847** | 4.577** | 4.129** | 4.064** | 4.393** |
| Vaccine brand:  CoronaVac-Sinovac |  | 0.906 |  |  |  |  |  |  |
| Pfizer |  | 0.449 |  |  |  |  |  |  |
| AstraZeneca  (Ref. group: Sputnik) |  | 0.960 |  |  |  |  |  |  |
| COVID-19 health concerns: Not worried (Ref. group: worried) |  |  | 2.674* |  |  |  |  |  |
| Self-reported health: Average/Poor (Ref. group: Good /Very good) |  |  |  | 1.795 |  |  |  |  |
| Comorbidities: at least 1 (Ref. group: None) |  |  |  |  | 1.038 |  |  |  |
| Depression: Ever diagnosed (Ref. group: Never) |  |  |  |  |  | 1.884 |  |  |
| Middle-low SES (Ref. group: Middle-high) |  |  |  |  |  |  | 1.735 |  |
| Food Insecurity (Ref. group: Food security) |  |  |  |  |  |  |  | 2.589* |
| Constant | 0.036** | 0.041** | 0.022** | 0.028** | 0.035** | 0.030** | 0.026** | 0.022** |

*NOTE*: Dependent variable in all models is COVID-19 vaccination (0=yes; 1=no). *Ref. group*, Reference group. ** p<0.01, * p<0.05, + p<0.1
